# HIV-Negative MSM Infected with Two Different Isolates of Drug-Resistant *Neisseria gonorrhoeae*—Case Report

**DOI:** 10.3390/pathogens13060497

**Published:** 2024-06-10

**Authors:** Martyna Biała, Bartosz Pencakowski, Beata Mączyńska, Konrad Starzyński, Bartosz Szetela

**Affiliations:** 1 Department of Infectious Diseases, Liver Diseases and Acquired Immune Deficiences, Wroclaw Medical University, 5 Koszarowa Street, 51-149 Wroclaw, Poland; 2 Department of Pharmaceutical Biology and Biotechnology, Wroclaw Medical University, 211 Borowska Street, 50-556 Wroclaw, Poland; 3 Department of Pharmaceutical Microbiology and Parasitology, Wroclaw Medical University, 211a Borowska Street, 50-556 Wroclaw, Poland; 4 All Saint’s Clinic, Wroclawskie Centrum Zdrowia SP ZOZ, 50-136 Wroclaw, Poland

**Keywords:** *Neisseria gonorrhoeae*, MSM, antibiotic resistance, STI

## Abstract

The antimicrobial resistance of *Neisseria gonorrhoeae* (NG) is an increasing public health concern, highlighted by the fact that gonococcus is considered as a ‘high’-priority pathogen by the WHO for research and development of new therapeutic options. According to the data of the European Centre for Disease Prevention and Control (ECDC) in 2022, the rate of NG infections is the highest recorded since European surveillance of sexually transmitted infections began in 2009. We report a brief description of a patient infected with two different isolates of drug-resistant *N. gonorrhoeae*. *N. gonorrhoeae* cultures were positive from oropharyngeal and urethral swabs and isolates had different antimicrobial susceptibility. We investigated the antimicrobial susceptibility of these isolates to six antimicrobials (ceftriaxone, cefixime, azithromycin, ciprofloxacin, tetracycline, and benzylpenicillin), and minimum inhibitory concentrations (MICs; mg/L) were determined using Etest on gonococcal isolates. Oropharyngeal isolate was resistant to azithromycin while urethral was resistant to penicillin, ciprofloxacin, and tetracycline. Two different and phylogenetically distinct sequence types of NG isolates were identified. Understanding the dynamics and drivers of resistance spread can provide an improved rationale for antibiotic management, and the level of NG resistance should be monitored closely.

## 1. Introduction

Gonorrhoea is a common sexually transmitted infection caused by the bacteria *Neisseria gonorrhoeae*. The number of gonorrhoea cases is highest among key populations such as men who have sex with men (MSM), sex workers, transgender women, and adolescents and young people in high-burden countries [1]. According to the data of the European Centre for Disease Prevention and Control (ECDC) in 2022, 70,881 confirmed *N. gonorrhoeae* infections were reported in 28 European Union/European Economic Area (EU/EEA) countries, with a crude notification rate of 17.9 cases per 100,000 population, indicating a 48% increase in the crude notification rate compared with 2021 and an 59% increase compared with 2018 [2]. The rate of *N. gonorrhoeae* infections is the highest recorded since European surveillance of sexually transmitted infections began in 2009 [2]. Resistance to azithromycin and ciprofloxacin was observed in 35% (7/20) and 50% (10/20) of NG isolates in Poland, respectively [3]. Furthermore, all of these NG isolates (20/20) were susceptible to ceftriaxone and cefixime [3].

Most bacterial STIs among MSM are asymptomatic, which makes diagnosis more challenging [4]. Therefore, there are recommendations for frequent asymptomatic triple-site screening for STIs in key populations [5].

There is also increasing evidence that antimicrobial use could be contributing to antimicrobial resistance and data indicate that drug-resistant *Neisseria gonorrhoeae* might be an increasing public health problem, highlighted by the fact that gonococcus is considered as a ‘high’-priority pathogen by the WHO for research and development of new antibiotics [6].

This is a brief description of a patient infected with two different isolates of drug-resistant *N. gonorrhoeae*.

## 2. Case Description

A 39-year-old MSM using HIV pre-exposure prophylaxis (PrEP) presented to our outpatient HIV/STI clinic with dysuria and urethral discharge lasting 7 days. Physical examination showed no other abnormalities. He reported having sexual contacts with three men, two from Poland and one from Spain. Cumulatively, 64% and 71% of his oral and anal sexual contacts, respectively, were reported as regularly protected by condoms. He denied substance use or chemsex. Oropharyngeal, urethral, and rectal swabs were collected for PCR and culture. We used Amies with Charcoal Transport Medium for swab culture. Swabs were inoculated into Thayer Martin, GC, and chocolate agar culture mediums and incubated in a 5% CO_2_ atmosphere. The identification of *N. gonorrhoeae* was performed according to API NH test (bioMerieux, Marcy-l’Étoile, France). *Neisseria gonorrhoeae* and *Chlamydia trachomatis* were detected by PCR. The patient’s therapy was initiated using ceftriaxone 1 g i.m. plus azithromycin 2 g p.o. as single doses as well as doxycycline 2 × 100 mg p.o. for 7 days. *N. gonorrhoeae* cultures were positive from oropharyngeal and urethral swabs and isolates had different antimicrobial susceptibility. We investigated the antimicrobial susceptibility of these isolates to six antimicrobials (ceftriaxone, cefixime, azithromycin, ciprofloxacin, tetracycline, and benzylpenicillin). Minimum inhibitory concentrations (MICs) were determined using Etest on gonococcal isolates. Oropharyngeal isolate was resistant to azithromycin (MIC = 2 mg/L) while urethral was resistant to penicillin (MIC = 8 mg/L), ciprofloxacin (MIC = 0.75 mg/L), and tetracycline (MIC = 8 mg/L).

### 2.1. Neisseria gonorrhoeae Genomic DNA Isolation and Molecular Typing (NG-MAST) Protocol

*Neisseria gonorrhoeae* multi-antigen sequence typing (NG-MAST) method was based on PCR amplification and sequence analysis of the gonococcal *porB* and *tbpB* genes, and subsequent characterization of specific Sequence Types (STs) of studied strains of the NG.

Bacterial DNA from all studied strains was extracted with a Bacterial & Yeast Genomic DNA Purification Kit (EURx, Gdansk, Poland). The assessment of gDNA quantity and quality was performed spectrophotometrically by NanoDrop™ 2000c spectrophotometer (Thermo Scientific™, Waltham, MA, USA).

PCR was performed with Hybrid DNA Polymerase Mastermix (EURx, Gdansk, Poland), and all reaction mixes were prepared according to the standard manufacturer’s protocol. For the amplification of *porB* and *tbpB* fragments, two previously designed pairs of primers were used: 5′-350CAA GAA GAC CTC GGC AA366-3′ (*por* forward) and 5′-1086CCG ACA ACC ACT TGG T1071-3′ (*por* reverse); and 5′-1098CGT TGT CGG CAG CGC GAA AAC1118-3′ (*tbpB* forward) and 5′-1686TTC ATC GGT GCG CTC GCC TTG1666-3′ (*tbpB* reverse), respectively [7]. Cycle sequencing was carried out with BrilliantDye™Terminator Cycle Sequencing Kit (NimaGen B.V., Nijmegen, The Netherlands) in twofold dilution of the reaction premix protocol in 10 µL according to the manufacturer’s instructions. Two sequencing reactions were conducted with each primer. Afterward, two reads were collected from each sequencing. Sequence Scanner Software v2.0 was used for the basecalling and all sequences were then analyzed and assembled with SnapGene Software (www.snapgene.com). Retrieved sequences for two studied strains collected from urethral swab and oropharyngeal swab (NG54U and NG54T, respectively) were uploaded to PubMLST.org database [8]. Obtained alleles, profiles, and isolates were subject to submission. Complete and characterized data of studied strains are shown in Table 1.

We have confirmed infection with two different strains of *Neisseria gonorrhoeae*.

### 2.2. Clinical Outcome

Complete recovery of the patient was achieved with resolved symptoms several days later.

## 3. Discussion

Some MSM are more likely to be diagnosed with *Neisseria gonorrhoea* than the general population due to risky sexual behaviors, i.e., multiple sex partners, casual sex partners, condomless sex, group sex, or chemsex [9]. Symptoms of gonorrhea vary by anatomical site in the general population: rectal and oropharyngeal infections are frequently asymptomatic, whereas a urethral infection is mainly symptomatic. Asymptomatic NG infection is especially frequent among MSM. Undetected and untreated, it may lead to the creation of a reservoir, which can result in onward transmission among multiple partners. It is estimated that approximately 80% of NG infections detected in MSM are asymptomatic and based on anatomical site; approximately 80–90% of oropharyngeal and rectal infections are asymptomatic [10,11,12]. Most guidelines state that sexually active MSM should undergo STI screening every 3–6 months, including three-site testing for *Chlamydia trachomatis* and *Neisseria gonorrhoeae*, and also serology of HIV and syphilis. The number of gonorrhoea cases reported each year continues to expand in the majority of EU/EEA countries in all groups—MSM, heterosexual men, and heterosexual women—and MSM accounted for the majority (60%) of gonorrhoea cases in the EU/EEA in 2022 [2].

We present a case of HIV-negative MSM diagnosed with asymptomatic oropharyngeal and symptomatic urethral NG infections. Kissing, oral sex without condoms, and saliva being used as lubricant may be responsible for the easy transmission of gonorrhoea. Even individuals with stable circuits of partners may be at an increased risk of NG infection and may introduce it to their sexual groups [13]. Moreover, this case highlights the importance of performing bacterial culture as the antimicrobial susceptibility patterns of isolates may differ at different anatomical sites.

To date, only a few similar reports have been described in the literature. Al-Madboly L. et al., described a case report of urethritis, conjunctivitis, and arthritis in a male patient infected with two different isolates of multiple drug-resistant *Neisseria gonorrhoeae* [14]. It revealed multiple drug resistances associated with β-lactamase production; only gentamicin, rifampicin, and azithromycin were active against the described pathogens. A dual therapy was initiated using gentamicin as well as azithromycin to treat the patient, and the symptoms resolved a week later [14]. We previously reported the case of a bisexual MSM infected with two new strains of drug-resistant *N. gonorrhoeae*—the rectal isolate was resistant to penicillin, ciprofloxacin, and tetracycline while the urethral isolate was resistant to ciprofloxacin [15]. The patient was treated with ceftriaxone and azithromycin as well as doxycycline (to treat the co-infection of *Chlamydia trachomatis*) and complete recovery was achieved [15]. The time of symptom resolution following effective treatment of gonorrhoea is variable, but in most patients symptoms resolve within several days. To minimize the spread of infection, persons treated for gonorrhea should be informed to abstain from sexual activity for 7 days after treatment and until all sex partners are treated [16].

According to the WHO, the antimicrobial resistance of *N. gonorrhoeae* has risen rapidly in recent years and has led to reduced therapeutic options [17]. Rising prevalence of azithromycin resistance remains a concern and may threaten the currently used dual-therapy regimen (ceftriaxone plus azithromycin) and high-dose ceftriaxone monotherapy adopted by some European countries, especially if ceftriaxone resistance starts to spread more widely [18]. Fortunately, till now NG strains resistant to ceftriaxone have only been sporadic and there have been no reports of sustained transmission of ceftriaxone-resistant *N. gonorrhoeae*. Hence, ceftriaxone remains an effective option for gonococcal infection.

## 4. Conclusions

Only early detection of emerging resistant strains and treatment, including enhanced focus on key populations, can reduce the impact of NG on public health. MSM are at greater risk for gonorrhoea. Understanding the dynamics and drivers of resistance spread can provide an improved rationale for antibiotic management and the level of NG resistance should be monitored closely.

## Figures and Tables

**Table 1:** Molecular characterization of studied *N. gonorrhoeae* strains.

N^o^	Isolate	PubMLST Isolate Id	*porB* Allele	*tbpB* Allele	Sequence Type
1	NG54U	141485	2596	2973	21218
2	NG54T	141486	6623	1867	21359

## Data Availability

The data presented in this study are available and can be shared on reasonable request sent to the corresponding author.
